# Ten Years of Landscape Genomics: Challenges and Opportunities

**DOI:** 10.3389/fpls.2017.02136

**Published:** 2017-12-12

**Authors:** Yong Li, Xue-Xia Zhang, Run-Li Mao, Jie Yang, Cai-Yun Miao, Zhuo Li, Ying-Xiong Qiu

**Affiliations:** ^1^College of Forestry, Henan Agricultural University, Zhengzhou, China; ^2^Key Laboratory of Conservation Biology for Endangered Wildlife of the Ministry of Education and Laboratory of Systematic and Evolutionary Botany and Biodiversity, College of Life Sciences, Zhejiang University, Hangzhou, China

**Keywords:** adaptive evolution, genetic structure, landscape genomics, molecular ecology, population genetics

## Abstract

Landscape genomics is a relatively new discipline that aims to reveal the relationship between adaptive genetic imprints in genomes and environmental heterogeneity among natural populations. Although the interest in landscape genomics has increased since this term was coined, studies on this topic remain scarce. Landscape genomics has become a powerful method to scan and determine the genes responsible for the complex adaptive evolution of species at population (mostly) and individual (more rarely) level. This review outlines the sampling strategies, molecular marker types and research categories in 37 articles published during the first 10 years of this field (i.e., 2007–2016). We also address major challenges and future directions for landscape genomics. This review aims to promote interest in conducting additional studies in landscape genomics.

## Introduction

Rapid global climate change is an important factor that affects biodiversity (Hoffmann and Sgrò, 2011). Adjusting their distribution range or local adaptation is the usual coping strategy of species toward rapid climate change (Aitken et al., 2008). Local adaptation requires the species to face long-term spatial environmental heterogeneity and eventually leads to adaptive differentiation of phenotypes. These changes might be due to phenotypic plasticity or heritable phenotypic variation. Exploring the adaptive evolution of species in response to spatial environmental heterogeneity will be useful in understanding initial adaptive divergence and evolutionary potential of a target species (Pluess et al., 2016). Landscape genomics is a powerful research field for investigating the adaptive evolution of species in response to spatial environmental heterogeneity (Vincent et al., 2013).

Joost et al. (2007) proposed landscape genomics as a relatively new discipline that aims to reveal the relationship between the adaptive genetic imprints in genomes and the environmental heterogeneity. Different from landscape genetics, landscape genomics requires a sufficient number of molecular markers to cover the entire genome. Emphasis is placed on adaptive evolution at the genome level (Miao et al., 2017). Landscape genetics, however, is biased toward using a relatively small number of molecular markers to reveal the relationship between environmental factors and the spatial genetic structure of populations (Dionne et al., 2008; Poelchau and Hamrick, 2012; Manel and Holderegger, 2013). Landscape genomic studies on many plant and animal species have been recently conducted (Berg et al., 2015; Manthey and Moyle, 2015; Leamy et al., 2016; Vangestel et al., 2016). These studies have achieved considerable progress on understanding of the relative roles of adaptive and non-adaptive processes in shaping patterns of genomic variation and the effects of environmental variables on adaptive differentiation at the genomic level. Although landscape genomics has been pursued for a decade, the studies, basic theoretical frameworks, and universal hypotheses in this field are still scarce. Thus, additional landscape genomic studies are needed to assist the construction of basic theoretical frameworks and formulation of universal hypotheses. This review summarizes the progress of landscape genomic studies such as conceptual and methodological developments as well as applied contributions during the previous decade. We then outline expected future directions in the field and encourage researchers to participate in this field.

## Methods

By searching the theme “landscape genomics” in the database of web of science^1^, and further looking through the related papers carefully, 37 articles focused on adaptive genetic imprints in genomes driven by environmental factors were finally selected. Supplementary Table S1 lists the molecular markers, sampling strategies, statistical methods, and research categories addressed in these articles.

## Sampling Strategies in Landscape Genomics

Sampling strategies in landscape genomics are divided into two major categories: random and stratified. The random sampling design includes scattered and clustered sampling. In the scattered sampling design, samples are randomly collected from across the species distribution range, while in the clustered sampling design, populations are divided into clusters according to environmental or genetic factors and samples are randomly taken from each cluster. A stratified sampling design can be performed to capture the range of variability across landscape variable(s) of interest. Thus, this sampling design requires a large amount of biological and environmental information of a target species. The optimal sampling scheme will be obtained by model calculation (Manel et al., 2012). The two sampling strategies mentioned above can be implemented at the individual or population level. The advantage of population sampling is more conducive to detect variation in gene frequency among populations than individual sampling. The most controversial topic in population sampling is multiple samples in fewer populations versus fewer samples in multiple populations. The former strategy is more representative in landscape genomic studies, but its accuracy in estimating genetic parameters is often questioned. In population genetics studies, the minimum sample size of a population should not be less than 20 individuals, 25–30 individuals are considered to be more reasonable (Hale et al., 2012). Therefore, it is necessary to ensure a minimum population sample size in the landscape genomic studies. Compared to population-based sampling, the application of individual sampling in landscape genomic studies is relatively scarce, but nonetheless suitable for clinal populations or those with unclear population structure (Jones et al., 2013).

## Molecular Markers in Landscape Genomics

Landscape genomic studies require molecular markers that are sufficiently spread throughout the genome (Balkenhol et al., 2009). However, most non-model species do not have established genomic information to appropriately place sufficient markers across the genome. Therefore, two characteristics, i.e., no requirement for *a priori* genome knowledge and a high covering density in genomes, are indispensable for the use of these molecular markers in landscape genomics (Yang et al., 2017).

Two types of molecular markers are suitable for landscape genomic studies. Type-I markers have no DNA sequence information, such as amplified fragment length polymorphisms, inter-simple sequence repeats, and start codon targeted polymorphisms. Type-II markers contain DNA sequence information, such as single-nucleotide polymorphisms (SNPs). Type-I markers require low generation cost but have few defects. Although these type-I markers may allow detecting loci potentially responsible for adaptation using outlier locus detection and environmental association analysis (EAA), the gene function of those loci cannot be easily validated, and thus might be false-positives. Type-II markers usually display high scanning density but have higher generation cost than type-I markers. However, type-II markers exhibit several advantages because these markers contain DNA sequence information. This information can help us annotate and map these markers on the genome. Based on the landscape genomic studies that we have selected (see Supplementary Table S1), SNP genotyping was mainly achieved through DNA microarrays. However, the use of DNA microarrays requires a large amount of prior gene information (Teng and Xiao, 2009). The recently developed reduced-representation genome sequencing (RRGS) is based on next-generation sequencing (NGS), which includes genotyping by sequencing (Elshire et al., 2011), restricted site associated DNA (Miller et al., 2007), and specific-locus amplified fragment sequencing (Sun et al., 2013). RRGS reduces the cost of sequencing, maintains high coverage of the genome, and does not require *a priori* genomic information. Thus, the use of RRGS is beneficial in landscape genomic research (Brauer et al., 2016). Most of the RRGS methods are currently based on Illumina sequencing platforms, which have an advantage of high accuracy and throughput and a disadvantage of short reading lengths. Third generation sequencing (TGS), such as MinION device by Oxford Nanopores and PacBio Sequel by Pacific BioSciences, has been recently developed to compensate for the short reading length of NGS. Although TGS maintains the speed and flux advantages of NGS, this method still exhibits some problems, such as high cost and error rate, which must be addressed (Mikheyev and Tin, 2014). In summary, the use of type-II markers to conduct landscape genomic studies can facilitate the indirect validation of loci potentially responsible for adaptation.

## Major Research Categories in Landscape Genomics

There are a wide variety of questions in ecology and evolution that can be addressed using a landscape genomic approach. We group these questions under two major research categories: (1) quantifying influence of spatial environmental variables on genomic divergence; (2) uncovering the environmental factors that shape adaptive genetic variation and the genetic basis of adaptive change.

## Quantifying Influence of Spatial Environmental Variables on Genomic Divergence

Isolation by distance (IBD) describes the local accumulation of genetic differences when dispersal between populations is geographically restricted (Slatkin, 1993). For IBD, gene flow path is assumed to be in a linear geographic distance. However, in natural landscapes, the paths of gene flow between populations are often non-linear and complex. In fact, populations that have identical habitats or small distances may also diverge when intervening landscape features inhibit dispersal between them (Isolation by Resistance, IBR; McRae, 2006; Ruiz-Gonzalez et al., 2015). Nevertheless, local genetic adaptation can also reduce gene flow among natural populations. This adaptive reduction in the effective rate of gene flow can contribute to a pattern of “isolation by environment” (IBE; Wang and Summers, 2010; Wang and Bradburd, 2014; Mosca et al., 2016). A strong pattern of IBE indicates that divergent selection is maintaining population differentiation in the face of possible dispersal (Schluter, 1998; Kawecki and Ebert, 2004). IBE can also arise from divergent habitat choice or other forms of biased dispersal (Armsworth and Roughgarden, 2008; Bolnick and Otto, 2013). Therefore, these different processes affect the spatial distribution of genetic variation and landscape genetic structure. Recently developed analytical methods can partition the often confounded patterns of IBD, IBE, and IBR when explaining genetic divergence across a landscape (Supplementary Table S1). The basic strategy is to use IBD as a null hypothesis against which IBE (or IBR) can be tested. Partial Mantel tests have been widely used in landscape genomics studies to evaluate the relative influence of different ecological and evolutionary factors on genetic differentiation. However, such tests have low statistical power and are prone to false positives (Guillot and Rousset, 2013). Recently, structural equation modelling (SEM) (Wang et al., 2013) and multiple matrix regression with randomization (MMRR) (Wang, 2013) have been used to quantitatively compare how much genetic divergence depends on IBD versus IBE (Zhang et al., 2016). In addition, the BEDASSLE package (Bradburd et al., 2013) is also used to estimate the relative contributions of IBD and IBE to genetic differentiation This Bayesian method models the allele frequencies in a set of populations at a set of unlinked loci as spatially correlated Gaussian processes, in which the covariance structure is a decreasing function of both geographic and ecological distance (Bradburd et al., 2013). In landscape genomic studies, multivariate statistical models are more appropriate when multidimensional niches are analyzed to identify ecological drivers of population genetic variation (Orsini et al., 2013). Redundancy analysis (RDA) (Legendre and Legendre, 2012) and canonical correlation analysis (CCA) (Parisod and Christin, 2008; Hecht et al., 2015) are commonly used to estimate the relative contribution of spatial and environmental variables. The CCA method can control for demographic effects if spatial autocorrelation is included in the model design, while RDA and partial RDA analyses are alternative and robust approaches that can control for spatial effects while analyzing others (Sork et al., 2013).

## Uncovering the Environmental Factors that Shape Adaptive Genetic Variation and the Genetic Basis of Adaptive Change

Polymorphic sites across species genomes will establish their adaptive differentiation to acclimatize to the heterogeneous environment. Landscape genomics attempts to detect these adaptive loci under selection and reveal potential environmental drivers of selection by using correlative methods. The detection of loci responsible for adaptation usually involves two steps. One is to detect the outlier loci; and the other is to associate the outlier loci with environment variables, referred to as EAA. The commonly used methods for detecting the outlier loci are ARLEQUIN (Excoffier et al., 2009), BAYESCAN (Foll and Gaggiotti, 2008), FLK (Bonhomme et al., 2010), and spatial ancestry analysis (SPA) (Yang et al., 2012). ARLEQUIN is applied to simulate a null distribution of *F*_ST_ values under a hierarchical island model, which is insensitive to the hierarchically subdivided population samples or those with a recently shared history. BAYESCAN is an *F*_ST_-based model to identify outlier loci according to Bayesian posterior probability. FLK deals with variation in effective population size and historical branching of populations by incorporating a population kinship matrix into the Lewontin and Krakauer (LK) statistic (Lewontin and Krakauer, 1973). SPA is a probabilistic model for the spatial structure of genetic variation that is used to identify loci showing extreme patterns of spatial differentiation. Compared with the two *F*_ST_-based approaches, SPA is particularly sensitive to strong spatial patterns in allele frequency and works at the individual level rather than at the population level. These methods are usually combined to distinguish the selected loci from the neutral loci and thus effectively reduce the false-positive rate (Wang et al., 2016). EAA, followed by outlier analysis, will be conducted to test whether these outlier loci are associated with particular environmental factors and under adaptive evolution.

The methods for conducting EAA can be divided into five broadly defined categories, including categorical tests, logistic regressions, matrix correlations, general linear models, and mixed effects models (Rellstab et al., 2015). A first category contains categorical tests, which compares allele frequencies of individuals or populations from different types of environments. The different types of environment are introduced as categorical variables in parametric or non-parametric tests. A second category comprises the statistical methods of logistic regressions, such as SAM, Samβada. The spatial analysis method (SAM; Joost et al., 2007) is the first implementation of logistic regression in EAA. SAM can compute multiple simultaneous univariate logistic regressions to test for association between allelic frequencies and environmental variables. However, this approach ignores neutral genetic structure, possibly leading to high false-positive rates under various demographic scenarios (De Mita et al., 2013; Frichot et al., 2013). Nevertheless, an extended version of SAM, Samβada (Joost et al., 2007) improves the performance of this method by adding neutral genetic structure as an additional factor (Rellstab et al., 2015). A third category contains a linear approach, matrix correlations, in which the effects of environmental factors and neutral genetic structure on allele frequencies are simultaneously estimated. The most widely used methods include a simple Mantel test and the partial Mantel test (Mantel, 1967). However, variations of the (partial) Mantel test may circumvent certain bias and autocorrelation problems (Legendre, 1993; Legendre et al., 2002). A fourth important category of statistical methods is general linear models in which a response variable is modeled as a linear function of some set of explanatory variables. The general linear model framework can be extended to models with multivariate response variables to account for the polygenic architecture of adaptive traits (Rellstab et al., 2015). The statistical methods include multiple linear regressions and univariate general linear models (Carl and Kuhn, 2007; Eckert et al., 2009) and canonical correlations and multivariate linear regressions, e.g., CCA (ter Braak and Smilauer, 2002; Legendre and Legendre, 2012) and RDA (Legendre and Legendre, 2012; Hecht et al., 2015). A fifth important category of statistical methods comprises the mixed effects models, such as BAYENV (Coop et al., 2010), LFMMs (Frichot et al., 2013), TASSEL (Bradbury et al., 2007), and EMMA (Kang et al., 2008). These approaches provide a unified statistical framework for controlling for the effects of neutral genetic structure (Rellstab et al., 2015). For example, BAYENV, based on a Bayesian generalized linear mixed model, is applied to test the correlation between allelic frequencies and environmental variables after correcting for population structure and size (Günther and Coop, 2013). Latent factor mixed models (LFMMs) implemented fast algorithms using a hierarchical Bayesian mixed model based on a variant of principal component analysis (PCA), in which the residual population structure is introduced via unobserved or latent factors (Frichot et al., 2013; Caye et al., 2016). In addition, a linear mixed-model method implemented in TASSEL (Bradbury et al., 2007) is used to identify candidate loci responsible for adaptation according to the association between the genotypes and climate variables (Yoder et al., 2014). Based on linear mixed models, Kang et al. (2008) developed an efficient mixed-model association (EMMA) method. As previously mentioned, in order to reduce the false-positive rate, it is desirable to combine more than two statistical methods to identify the environment-associated loci (Yang et al., 2017).

## Major Challenges

Although great progress in landscape genomics has been achieved in the past decade, two major challenges remain to be solved in the future. One is the presence of false positives, which have been a major problem in landscape genomics because of the lack of validation for adaptive loci. Three solutions will help solve this major challenge. First, robust detection methods must be developed, and multiple detection methods must be used to reduce the false-positive rates. Second, type-II markers that contain DNA sequence information must be selected. Although type-I markers may allow detecting loci potentially responsible for adaptation, the gene function of these detected loci are difficult to be validated. Type-II markers have DNA sequence information, which can be indirectly validated through the annotation of gene function. Third, a part of the loci responsible for adaptation must be validated using gene transfer and gene knockout technologies. Since most of previous landscape genomics studies have focused on non-model species, the detected loci responsible for adaptation do not have functional verification. Thus, in future, more experiments are needed to validate the function and adaptive generality of the detecting loci responsible for adaptation. In addition, most previous studies have showed great concern on gene differentiation rather than phenotypic differentiation (Manthey and Moyle, 2015; Di Pierro et al., 2016). The acquisition of adaptive phenotypic data has been conducted in a few recent landscape genomic studies (De Kort et al., 2014; Roschanski et al., 2016). Thus, obtaining the phenotypic data through common garden experiments and reciprocal transplant experiments should be considered in future.

## Recommendations for Future Research

The present landscape genomics mainly addresses two issues, i.e., influence of spatial environmental variables on genomic divergence and effects of the environmental factors on adaptive genetic variation. The following concerns need to be addressed in landscape genomic studies. (1) Previous studies have determined the specific genes that undergo adaptive changes and the environmental factors that contribute to these changes. However, the specific reason why these particular genes or environmental variables exhibit these functions remains unknown. (2) Type-II markers can help us reveal these specific genes. However, the metabolic pathways of the involved genes and the adaptive phenotypes controlled by these genes need to be identified. (3) Regional species in extreme environments usually establish some convergent adaptive changes in their genes or phenotypes. However, most regional species not living in extreme environments have various adaptive differentiations. Thus, the commonalities behind these diverse adaptive differentiations must be determined. (4) The distribution range of species and their ability to respond to climate change largely depend on their landscape adaptability, which is usually determined by the potential adaptive differentiation of the genome and the gene dispersal ability of the species. Thus, a landscape adaptation index must be established to measure the adaptability of species. In summary, landscape genomics is an efficient method to study the adaptive evolution of species. We hope that this review of studies on landscape genomics over the past 10 years will assist in promoting future research in this field.

## Author Contributions

X-XZ and R-LM wrote the original draft of article; JY, C-YM, and ZL revised this article; Y-XQ and YL conceived the ideas and contributed to substantial revisions; all authors read and approved the final version of the manuscript.

## Conflict of Interest Statement

The authors declare that the research was conducted in the absence of any commercial or financial relationships that could be construed as a potential conflict of interest.

## Footnotes

1 http://www.isiknowledge.com/

## References

[B1] Aitken S. N. Yeaman S. Holliday J. A. Wang T. L. Curtis-McLane S. (2008). Adaptation, migration or extirpation: climate change outcomes for tree populations. *Evol. Appl.* 1 95–111. 10.1111/j.1752-4571.2007.00013.x 25567494 PMC3352395

[B2] Armsworth P. R. Roughgarden J. E. (2008). The structure of clines with fitness-dependent dispersal. *Am. Nat.* 172 648–657. 10.1086/591685 18828744

[B3] Balkenhol N. Waits L. P. Dezzani R. J. (2009). Statistical approaches in landscape genetics: an evaluation of methods for linking landscape and genetic data. *Ecography* 32 818–830. 10.1111/j.1600-0587.2009.05807.x

[B4] Berg P. R. Jentoft S. Star B. Ring K. H. Knutsen H. Lien S. (2015). Adaptation to low salinity promotes genomic divergence in Atlantic Cod (*Gadus morhua* L.). *Genome Biol. Evol.* 7 1644–1663. 10.1093/gbe/evv093 25994933 PMC4494048

[B5] Bolnick D. I. Otto S. P. (2013). The magnitude of local adaptation under genotype-dependent dispersal. *Ecol. Evol.* 3 4722–4735. 10.1002/ece3.850 24363900 PMC3867907

[B6] Bonhomme M. Chevalet C. Servin B. Boitard S. Abdallah J. Blott S. (2010). Detecting selection in population trees: the Lewontin and Krakauer test extended. *Genetics* 186 241–262. 10.1534/genetics.104.117275 20855576 PMC2940290

[B7] Bradburd G. S. Ralph P. L. Coop G. M. (2013). Disentangling the effects of geographic and ecological isolation on genetic differentiation. *Evolution* 67 3258–3273. 10.1111/evo.12193 24102455 PMC3808528

[B8] Bradbury P. J. Zhang Z. Kroon D. E. Casstevens T. M. Ramdoss Y. Buckler E. S. (2007). TASSEL: software for association mapping of complex traits in diverse samples. *Bioinformatics* 23 2633–2635. 10.1093/bioinformatics/btm308 17586829

[B9] Brauer C. J. Hammer M. P. Beheregaray L. B. (2016). Riverscape genomics of a threatened fish across a hydroclimatically heterogeneous river basin. *Mol. Ecol.* 25 5093–5113. 10.1111/mec.13830 27575282

[B10] Carl G. Kuhn I. (2007). Analyzing spatial autocorrelation in species distributions using Gaussian and logit models. *Ecol. Model.* 207 159–170. 10.1016/j.ecolmodel.2007.04.024

[B11] Caye K. Deist T. M. Martins H. Michel O. François O. (2016). TESS3: fast inference of spatial population structure and genome scans for selection. *Mol. Ecol. Resour.* 16 540–548. 10.1111/1755-0998.12471 26417651

[B12] Coop G. Witonsky D. Di Rienzo A. Pritchard J. K. (2010). Using environmental correlations to identify loci underlying local adaptation. *Genetics* 185 1411–1423. 10.1534/genetics.110.114819 20516501 PMC2927766

[B13] De Kort H. Vandepitte K. Bruun H. H. Closset-Kopp D. Honnay O. Mergeay J. (2014). Landscape genomics and a common garden trial reveal adaptive differentiation to temperature across Europe in the tree species *Alnus glutinosa*. *Mol. Ecol.* 23 4709–4721. 10.1111/mec.12813 24860941

[B14] De Mita S. Thuillet A. C. Gay L. Ahmadi N. Manel S. Ronfort J. (2013). Detecting selection along environmental gradients: analysis of eight methods and their effectiveness for outbreeding and selfing populations. *Mol. Ecol.* 22 1383–1399. 10.1111/mec.12182 23294205

[B15] Di Pierro E. A. Mosca E. Rocchini D. Binelli G. Neale D. B. La Porta N. (2016). Climate-related adaptive genetic variation and population structure in natural stands of Norway spruce in the South-Eastern Alps. *Tree Genet. Genomes* 12:16 10.1007/s11295-016-0972-4

[B16] Dionne M. Caron F. Dodson J. J. Bernatchez L. (2008). Landscape genetics and hierarchical genetic structure in Atlantic salmon: the interaction of gene flow and local adaptation. *Mol. Ecol.* 17 2382–2396. 10.1111/j.1365-294X.2008.03771.x 18430145

[B17] Eckert A. J. Pande B. Ersoz E. S. Wright M. H. Rashbrook V. K. Nicolet C. M. (2009). High-throughput genotyping and mapping of single nucleotide polymorphisms in loblolly pine (*Pinus taeda* L.). *Tree Genet. Genomes* 5 225–234. 10.1007/s11295-008-0183-8 24192835

[B18] Elshire R. J. Glaubitz J. C. Sun Q. Poland J. A. Kawamoto K. Buckler E. S. (2011). A robust, simple Genotyping-By-Sequencing (GBS) approach for high diversity species. *PLOS ONE* 6:e19379. 10.1371/journal.pone.0019379 21573248 PMC3087801

[B19] Excoffier L. Hofer T. Foll M. (2009). Detecting loci under selection in a hierarchically structured population. *Heredity* 103 285–298. 10.1038/hdy.2009.74 19623208

[B20] Foll M. Gaggiotti O. (2008). A genome scan method to identify selected loci appropriate for both dominant and codominant markers: a Bayesian perspective. *Genetics* 180 977–993. 10.1534/genetics.108.092221 18780740 PMC2567396

[B21] Frichot E. Schoville S. D. Bouchard G. François O. (2013). Testing for associations between loci and environmental gradients using latent factor mixed models. *Mol. Biol. Evol.* 30 1687–1699. 10.1093/molbev/mst063 23543094 PMC3684853

[B22] Guillot G. Rousset F. (2013). Dismantling the Mantel tests. *Methods Ecol. Evol.* 4 336–344. 10.1111/2041-210x.12018

[B23] Günther T. Coop G. (2013). Robust identification of local adaptation from allele frequencies. *Genetics* 195 205–220. 10.1534/genetics.113.152462 23821598 PMC3761302

[B24] Hale M. L. Burg T. M. Steeves T. E. (2012). Sampling for microsatellite-based population genetic studies: 25 to 30 individuals per population is enough to accurately estimate allele frequencies. *PLOS ONE* 7:e45170. 10.1371/journal.pone.0045170 22984627 PMC3440332

[B25] Hecht B. C. Matala A. P. Hess J. E. Narum S. R. (2015). Environmental adaptation in Chinook salmon (*Oncorhynchus tshawytscha*) throughout their North American range. *Mol. Ecol.* 24 5573–5595. 10.1111/mec.13409 26465117

[B26] Hoffmann A. A. Sgrò C. M. (2011). Climate change and evolutionary adaptation. *Nature* 470 479–485. 10.1038/nature09670 21350480

[B27] Jones M. R. Forester B. R. Teufel A. I. Adams R. V. Anstett D. N. Goodrich B. A. (2013). Integrating landscape genomics and spatially explicit approaches to detect loci under selection in clinal populations. *Evolution* 67 3455–3468. 10.1111/evo.12237 24299400

[B28] Joost S. Bonin A. Bruford M. W. Després L. Conord C. Erhardt G. (2007). A spatial analysis method (SAM) to detect candidate loci for selection: towards a landscape genomics approach to adaptation. *Mol. Ecol.* 16 3955–3969. 10.1111/j.1365-294X.2007.03442.x 17850556

[B29] Kang H. M. Zaitlen N. A. Wade C. M. Kirby A. Heckerman D. Daly M. J. (2008). Efficient control of population structure in model organism association mapping. *Genetics* 178 1709–1723. 10.1534/genetics.107.080101 18385116 PMC2278096

[B30] Kawecki T. J. Ebert D. (2004). Conceptual issues in local adaptation. *Ecol. Lett.* 7 1225–1241. 10.1111/j.1461-0248.2004.00684.x

[B31] Leamy L. J. Lee C. R. Song Q. J. Mujacic I. Luo Y. Chen C. Y. (2016). Environmental versus geographical effects on genomic variation in wild soybean (*Glycine soja*) across its native range in northeast Asia. *Ecol. Evol.* 6 6332–6344. 10.1002/ece3.2351 27648247 PMC5016653

[B32] Legendre P. (1993). Spatial autocorrelation: trouble or new paradigm? *Ecology* 74 1659–1673. 10.2307/1939924

[B33] Legendre P. Dale M. R. T. Fortin M. J. Gurevitch J. Hohn M. Myers D. (2002). The consequences of spatial structure for the design and analysis of ecological field surveys. *Ecography* 25 601–615. 10.1034/j.1600-0587.2002.250508.x

[B34] Legendre P. Legendre L. (2012). *Numerical Ecology.* Oxford: Elsevier.

[B35] Lewontin R. C. Krakauer J. (1973). Distribution of gene frequency as a test of the theory of the selective neutrality of polymorphisms. *Genetics* 74 175–195. 4711903 10.1093/genetics/74.1.175 PMC1212935

[B36] Manel S. Albert C. H. Yoccoz N. G. (2012). “Sampling in landscape genomics,” in *Data Production and Analysis in Population Genomics*, eds Pompanon F. Bonin A. (New York, NY: Humana Press), 3–12. 10.1007/978-1-61779-870-2_1 22665272

[B37] Manel S. Holderegger R. (2013). Ten years of landscape genetics. *Trends Ecol. Evol.* 28 614–621. 10.1016/j.tree.2013.05.012 23769416

[B38] Mantel N. (1967). The detection of disease clustering and a generalized regression approach. *Cancer Res.* 27 209–220. 6018555

[B39] Manthey J. D. Moyle R. G. (2015). Isolation by environment in white-breasted Nuthatches (*Sitta carolinensis*) of the Madrean Archipelago sky islands: a landscape genomics approach. *Mol. Ecol.* 24 3628–3638. 10.1111/mec.13258 26037653

[B40] McRae B. H. (2006). Isolation by resistance. *Evolution* 60 1551–1561. 10.1111/j.0014-3820.2006.tb00500.x 17017056

[B41] Miao C. Y. Li Y. Yang J. Mao R. L. (2017). Landscape genomics reveal that ecological character determines adaptation: a case study in smoke tree (*Cotinus coggygria* Scop.). *BMC Evol. Biol.* 17:202. 10.1186/s12862-017-1055-3 28835216 PMC5569454

[B42] Mikheyev A. S. Tin M. M. Y. (2014). A first look at the Oxford Nanopore MinION sequencer. *Mol. Ecol. Resour.* 14 1097–1102. 10.1111/1755-0998.12324 25187008

[B43] Miller M. R. Dunham J. P. Amores A. Cresko W. A. Johnson E. A. (2007). Rapid and cost-effective polymorphism identification and genotyping using restriction site associated DNA (RAD) markers. *Genome Res.* 17 240–248. 10.1101/gr.5681207 17189378 PMC1781356

[B44] Mosca E. Gugerli F. Eckert A. J. Neale D. B. (2016). Signatures of natural selection on *Pinus cembra*, and P. mugo, along elevational gradients in the Alps. *Tree Genet. Genomes* 12:9 10.1007/s11295-015-0964-9

[B45] Orsini L. Mergeay J. Vanoverbeke J. De Meester L. (2013). The role of selection in driving landscape genomic structure of the waterflea *Daphnia magna*. *Mol. Ecol.* 22 583–601. 10.1111/mec.12117 23174029

[B46] Parisod C. Christin P. A. (2008). Genome-wide association to fine-scale ecological heterogeneity within a continuous population of *Biscutella laevigata* (Brassicaceae). *New Phytol.* 178 436–447. 10.1111/j.1469-8137.2007.02361.x 18208468

[B47] Pluess A. R. Frank A. Heiri C. Lalagüe H. Vendramin G. G. Oddou-Muratorio S. (2016). Genome-environment association study suggests local adaptation to climate at the regional scale in *Fagus sylvatica*. *New Phytol.* 210 589–601. 10.1111/nph.13809 26777878

[B48] Poelchau M. F. Hamrick J. L. (2012). Differential effects of landscape-level environmental features on genetic structure in three codistributed tree species in Central America. *Mol. Ecol.* 21 4970–4982. 10.1111/j.1365-294X.2012.05755.x 22988889

[B49] Rellstab C. Gugerli F. Eckert A. J. Hancock A. M. Holderegger R. (2015). A practical guide to environmental association analysis in landscape genomics. *Mol. Ecol.* 24 4348–4370. 10.1111/mec.13322 26184487

[B50] Roschanski A. M. Csilléry K. Liepelt S. Oddou-Muratorio S. Ziegenhagen B. Huard F. (2016). Evidence of divergent selection for drought and cold tolerance at landscape and local scales in *Abies alba* Mill. in the French Mediterranean Alps. *Mol. Ecol.* 25 776–794. 10.1111/mec.13516 26676992

[B51] Ruiz-Gonzalez A. Cushman S. A. Madeira M. J. Randi E. Gómez-Moliner B. J. (2015). Isolation by distance, resistance and/ or clusters? lessons learned from a forest-dwelling carnivore inhabiting a heterogeneous landscape. *Mol. Ecol.* 24 5110–5129. 10.1111/mec.13392 26394893

[B52] Schluter D. (1998). “Ecological causes of speciation,” in *Endless Forms: Species and Speciation* eds Howard D. J. Berlochers S. H. (Oxford: Oxford University Press) 114–129.

[B53] Slatkin M. (1993). Isolation by distance in equilibrium and nonequilibrium populations. *Evolution* 47 264–279. 10.1111/j.1558-5646.1993.tb01215.x 28568097

[B54] Sork V. L. Aitken S. N. Dyer R. J. Eckert A. J. Legendre P. Neale D. B. (2013). Putting the landscape into the genomics of trees: approaches for understanding local adaptation and population responses to changing climate. *Tree Genet. Genomes* 9 901–911. 10.1007/s11295-013-0596-x

[B55] Sun X. Liu D. Zhang X. Li W. Liu H. Hong W. (2013). SLAF-seq: an efficient method of large-scale de novo SNP discovery and genotyping using high-throughput sequencing. *PLOS ONE* 8:e58700. 10.1371/journal.pone.0058700 23527008 PMC3602454

[B56] Teng X. K. Xiao H. S. (2009). Perspectives of DNA microarray and next-generation DNA sequencing technologies. *Sci. China Life Sci.* 52 7–16. 10.1007/s11427-009-0012-9 19152079

[B57] ter Braak C. J. F. Smilauer P. (2002). *CANOCO Reference Manual and CanoDraw for Windows User’s Guide: Software for Canonical Community Ordination (version 4.5).* New York, NY: Microcomputer Power.

[B58] Vangestel C. Vázquez-Lobo A. Martínez-García P. J. Calic I. Wegrzyn J. L. Neale D. B. (2016). Patterns of neutral and adaptive genetic diversity across the natural range of sugar pine (*Pinus lambertiana*, Dougl.). *Tree Genet.Genomes* 12:15 10.1007/s11295-016-0998-7

[B59] Vincent B. Dionne M. Kent M. P. Lien S. Bernatchez L. (2013). Landscape genomics in Atlantic salmon (*Salmo salar*): searching for gene-environment interactions driving local adaptation. *Evolution* 67 3469–3487. 10.1111/evo.12139 24299401

[B60] Wang I. J. (2013). Examining the full effects of landscape heterogeneity on spatial genetic variation: a multiple matrix regression approach for quantifying geographic and ecological isolation. *Evolution* 67 3403–3411. 10.1111/evo.12134 24299396

[B61] Wang I. J. Bradburd G. S. (2014). Isolation by environment. *Mol. Ecol.* 23 5649–5662. 10.1111/mec.12938 25256562

[B62] Wang I. J. Glor R. E. Losos J. B. (2013). Quantifying the roles of ecology and geography in spatial genetic divergence. *Ecol. Lett.* 16 175–182. 10.1111/ele.12025 23137142

[B63] Wang I. J. Summers K. (2010). Genetic structure is correlated with phenotypic divergence rather than geographic isolation in the highly polymorphic strawberry poison-dart frog. *Mol. Ecol.* 19 447–458. 10.1111/j.1365-294X.2009.04465.x 20025652

[B64] Wang T. Wang Z. Xia F. Su Y. J. (2016). Local adaptation to temperature and precipitation in naturally fragmented populations of *Cephalotaxus oliveri*, an endangered conifer endemic to China. *Sci. Rep.* 6:25031. 10.1038/srep25031 27113970 PMC4844950

[B65] Yang J. Miao C. Y. Mao R. L. Li Y. (2017). Landscape population genomics of forsythia (*Forsythia suspensa*) reveal that ecological habitats determine the adaptive evolution of species. *Front. Plant Sci.* 8:481. 10.3389/fpls.2017.00481 28424728 PMC5380681

[B66] Yang W. Y. Novembre J. Eskin E. Halperin E. (2012). A model-based approach for analysis of spatial structure in genetic data. *Nat. Genet.* 44 725–731. 10.1038/ng.2285 22610118 PMC3592563

[B67] Yoder J. B. Stanton-Geddes J. Zhou P. Briskine R. Young N. D. Tiffin P. (2014). Genomic signature of adaptation to climate in *Medicago truncatula*. *Genetics* 196 1263–1275. 10.1534/genetics.113.159319 24443444 PMC3982686

[B68] Zhang Y. H. Wang I. J. Comes H. P. Peng H. Qiu Y. X. (2016). Contributions of historical and contemporary geographic and environmental factors to phylogeographic structure in a Tertiary relict species, *Emmenopterys henryi* (Rubiaceae). *Sci. Rep.* 6:24041. 10.1038/srep24041 27137438 PMC4853719

