# Supplementary material for: Ten Years of Landscape Genomics: Challenges and Opportunities
Source: Front Plant Sci. 2017 Dec 12;8:2136. doi: 10.3389/fpls.2017.02136 (PMC5733015; doi:10.3389/fpls.2017.02136)
Supplement: Supplementary file 1 [file Table_1.DOCX]

Supplementary Material

Ten years of landscape genomics: challenges and opportunities

**Yong Li^1^, Xue-Xia Zhang^1^, Run-Li Mao^1^, Jie Yang^1^, Cai-Yun Miao^1^, Zhuo Li^1^ and Ying-Xiong Qiu^2^***

*** Correspondence:** Ying-Xiong Qiu: qyxhero@zju.edu.cn

# 1 Supplementary Table

**Table S1 | Review of 37 articles in landscape genomics from 2007 to 2016.**

| No. | Year | Title | Molecular Marker | Sampling strategy | Sequencing Technology | Statistical methods | Research Category |  |
| --- | --- | --- | --- | --- | --- | --- | --- | --- |
| 1 | 2007 | A spatial analysis method (SAM) to detect candidate loci for selection: towards a landscape genomics approach to adaptation. | SSRs and AFLPs | Random sampling at population level | Not involve | SAM, DFIST, FDIST2 | (2) |  |
| 2 | 2008 | Genome-wide association to fine-scale ecological heterogeneity within a continuous population of Biscutella laevigata (Brassicaceae) | AFLPs | Random sampling at population level | Not involve | CCA, partial Mantel tests | (1) |  |
| 3 | 2009 | Landscape genomics and biased FST approaches reveal single nucleotide polymorphisms under selection in goat breeds of North-East Mediterranean | SNPs | Random sampling at population level | PCR-RFLP, SSCP, SnaPshot, or Taqman | SAM, FDIST2 | (2) |  |
| 4 | 2010 | Back to nature: ecological genomics of loblolly pine (Pinus taeda, Pinaceae) | SNPs | Random sampling at population level | Illumina infinium platform | Bayesian geographical analysis | (2) |  |
| 5 | 2010 | Tracking genes of ecological relevance using a genome scan in two independent regional population samples of Arabis alpina | AFLPs | Random sampling at population level | Not involve | GEE | (2) |  |
| 6 | 2012 | Environmental adaptation contributes to gene polymorphism across the Arabidopsis thaliana genome | SNPs | Random sampling at individual level | Illumina sequencing platform | PCA, CCA, partial Mantel tests | (2) |  |
| 7 | 2012 | Local adaptation in the flowering-time gene network of balsam poplar, Populus balsamifera L. | SNPs | Random sampling at population level | Sequenom MassARRAY platform | BayeScan, BAYENV, FDIST2 | (2) |  |
| 8 | 2013 | Integrating landscape genomics and spatially explicit approaches to detect loci under selection in clinal populations | AFLPs | Stratified sampling at individual level | Not involve | GLMs, GLMMs, GAMMs, DFDIST, BayeScan | (2) |  |
| 9 | 2013 | Landscape genomics in Atlantic salmon (Salmo salar): searching for gene-environment interactions driving local adaptation. | SNPs | Random sampling at population level | CIGENE SNP array platform | RDA | (1) |  |
| 10 | 2013 | The role of selection in driving landscape genomic structure of the waterflea Daphnia magna | SSRs and SNPs | Random sampling at population level | Sequenom MassARRAY platform | RDA, partial Mantel tests | (2) |  |
| 11 | 2014 | Adaptations to Climate-Mediated Selective Pressures in Sheep | SNPs | Random sampling at population level | ISGC SNP50K BeadChip platform | FDIST2, SAM, LFMM | (2) |  |
| 12 | 2014 | Landscape genomics of Populus trichocarpa: the role of hybridization, limited gene flow, and natural selection in shaping patterns of population structure | SNPs | Random sampling at population level | Infinium II assay platform | FDIST2, BayeScan, Bayenv | (2) |  |
| 13 | 2014 | Landscape genomics and a common garden trial reveal adaptive differentiation to temperature across Europe in the tree species Alnus glutinosa | SNPs | Random sampling at population level | Genotyping by sequencing Illumina HiSeq 2000 platform | BayeScan, LFMM | (2) |  |
| 14 | 2014 | Assessing The spatial dependence of adaptive loci in 43 European and Western Asian goat breeds using AFLP markers | AFLPs | Random sampling at population level | Not involve | SAM, DFDIST, BayeScan | (2) |  |
| 15 | 2015 | Landscape genomics of Sphaeralcea ambigua in the Mojave Desert: a multivariate, spatially-explicit approach to guide ecological restoration | AFLPs | Random sampling at population level | Not involve | BayeScan, GLMs | (2) |  |
| 16 | 2015 | Environmental adaptation in Chinook salmon (Oncorhynchus tshawytscha) throughout their North American range | SNPs | Random sampling at population level | Restriction-site associated DNA sequencing Illumina platform | FDIST2, RDA, | (2) |  |
| 17 | 2015 | Landscape genomics reveal signatures of local adaptation in barley (Hordeum vulgare L.) | SNPs | Random sampling at individual level | Genotyping by sequencing Illumina HiSeq 2000 platform | Bayenv, LFMM, RDA | (2) |  |
| 18 | 2015 | Genome-wide analyses suggest parallel selection for universal traits may eclipse local environmental selection in a highly mobile carnivore | SNPs | Random sampling at population level | Illumina CanineHD BeadChip microarray platform | BayeScan, SAM | (2) |  |
| 19 | 2015 | Isolation by environment in White-breasted Nuthatches (Sitta carolinensis) of the Madrean Archipelago sky islands: a landscape genomics approach | SNPs | Random sampling at population level | Restriction site associated DNA sequencing Illumina HiSeq 2500 platform | partial Mantel tests, BayeScan, Bayenv, LFMM | (2) |  |
| 20 | 2015 | Adaptation to low salinity promotes genomic divergence in Atlantic cod (Gadus morhua L.) | SNPs | Random sampling at population level | Illumina SNP chip platform | BayeScan, FDIST2, Bayenv | (2) |  |
| 21 | 2015 | Sun skink landscape genomics: assessing the roles of micro-evolutionary processes in shaping genetic and phenotypic diversity across a heterogeneous and fragmented landscape | SNPs | Random sampling at population level | Restriction site associated DNA sequencing Illumina HiSeq 2500 platform | partial Mantel tests | (1) |  |
| 22 | 2015 | Combining niche modelling and landscape genetics to study local adaptation: A novel approach illustrated using alpine plants | AFLPs | Random sampling at population level | Not involve | GEE | (2) |  |
| 23 | 2015 | Ecological genomics meets community-level modelling of biodiversity: mapping the genomic landscape of current and future environmental adaptation | SNPs | Random sampling at population level | Sequenom MassARRAY platform | GDM, the hierarchical model in ARLEQUIN, BayeScan, Bayenv | (2) |  |
| 24 | 2016 | Landscape genomics reveals altered genome wide diversity within revegetated stands of Eucalyptus microcarpa (Grey Box) | SNPs | Random sampling at population level | Diversity Arrays Technology Illumina seqencing platform | PCA, IBD | (2) |  |
| 25 | 2016 | Signatures of local adaptation in candidate genes of oaks (Quercus spp.) with respect to present and future climatic conditions | SSRs and SNPs | Random sampling at population level | Illumina MiSeq platform | LFMM | (2) |  |
| 26 | 2016 | Genome scan reveals selection acting on genes linked to stress response in wild pearl millet | SNPs | Random sampling at population level | Illumina HighSeq 2000 platform | PCA, F ST approach, BayeScan, Bayenv | (2) |  |
| 27 | 2016 | Seascape genomics provides evidence for thermal adaptation and current-mediated population structure in American lobster (Homarus americanus) | SNPs | Random sampling at population level | Restriction-site associated DNA sequencing Illumina platform | the infinite island model in ARLEQUIN, BayeScan, Outflank, Pearson correlation tests, Bayenv, LFMM, RDA | (2) |  |
| 28 | 2016 | Riverscape genomics of a threatened fish across a hydroclimatically heterogeneous river basin | SNPs | Random sampling at population level | Restriction-site associated DNA sequencing Illumina HiSeq 2000 platform | the hierarchical model in ARLEQUIN, BayeScan, GLMMs, RDA | (2) |  |
| 29 | 2016 | Environmental versus geographical effects on genomic variation in wild soybean (Glycine soja) across its native range in northeast Asia | SNPs | Random sampling at individual level | Illumina infinium platform | CCA, RDA | (1) |  |
| 30 | 2016 | SNP discovery in candidate adaptive genes using exon capture in a free-ranging alpine ungulate | SNPs | Random sampling at population level | microfluidic  qPCR-based SNP chip | FDIST2, BayeScan, SAM | (2) |  |
| 31 | 2016 | Patterns of neutral and adaptive genetic diversity across the natural range of sugar pine (Pinus lambertiana Dougl.) | SNPs | Random sampling at population level | Illumina GoldenGate SNP genotyping platform | Bayenv, LFMM | (2) |  |
| 32 | 2016 | Genome-environment association study suggests local adaptation to climate at the regional scale in Fagus sylvatica | SSRs and SNPs | Stratified sampling at population level | KASP genotyping technology | LFMM | (2) |  |
| 33 | 2016 | Climate-related adaptive genetic variation and population structure in natural stands of Norway spruce in the South-Eastern Alps | SNPs | Random sampling at population level | Illumina GoldenGate SNP genotyping platform | BayeScan, Bayenv | (2) |  |
| 34 | 2016 | Signatures of natural selection on Pinus cembra and P. mugo along elevational gradients in the Alps | SNPs | Random sampling at population level | Illumina GoldenGate SNP genotyping | BayeScan, Bayenv, LFMM | (2) |  |
| 35 | 2016 | Climate variables explain neutral and adaptive variation within salmonid metapopulations: the importance of replication in landscape genetics | SNPs | Random sampling at population level | ABI Taqman  assays platform | GESTE, DISTLM | (2) |  |
| 36 | 2016 | Evidence of divergent selection for drought and cold tolerance at landscape and local scales in Abies alba Mill. in the French Mediterranean Alps | SNPs | Random sampling at population level | KASP genotyping technology | HFDIST, BayeScan, FLK, LFMM | (2) |  |
| 37 | 2016 | Contributions of historical and contemporary geographic and environmental factors to phylogeographic structure in a Tertiary relict species, *Emmenopterys henryi* (Rubiaceae) | AFLPs | Random sampling at population level | Not involve | IBD, IBE, MMRR, SEM | (1) |  |
| Research Category: (1) quantifying influence of spatial environmental variables on genomic divergence; (2) uncovering the environmental factors that shape adaptive genetic variation and the genetic basis of adaptive change. | | | | | | | | |

**References**

Abebe, T.D., Naz, A.A., and Léon, J. (2015). Landscape genomics reveal signatures of local adaptation in barley (*Hordeum vulgare* L.). *Front. Plant Sci.* 6, 813*.*

Barley, A.J., Monnahan, P.J., Thomson, R.C., Grismer, L.L., and Brown, R.M. (2015). Sun skink landscape genomics: assessing the roles of micro-evolutionary processes in shaping genetic and phenotypic diversity across a heterogeneous and fragmented landscape. *Mol. Ecol.* 24, 1696-1712.

Benestan, L., Quinn, B.K., Maaroufi, H., Laporte, M., Clark, F.K., Greenwood, S.J., Rochette, R., and Bernatchez, L. (2016). Seascape genomics provides evidence for thermal adaptation and current-mediated population structure in american lobster (*Homarus americanus*). *Mol. Ecol.* 25, 5073-5092.

Berg, P.R., Jentoft, S., Star, B., Ring, K.H., Knutsen, H., Lien, S., Jakobsen, K.S., and Andre, C. (2015). Adaptation to low salinity promotes genomic divergence in Atlantic Cod (*Gadus morhua* L*.*). *Genome Biol. Evol.* 7, 1644-1663.

Berthouly-Salazar, C., Thuillet, A.C., Rhoné, B., Mariac, C., Ousseini, I.S., Couderc, M., Tenaillon, M.I., and Vigouroux, Y. (2016). Genome scan reveals selection acting on genes linked to stress response in wild pearl millet. *Mol. Ecol.* 25, 5500-5512.

Brauer, C.J., Hammer, M.P., and Beheregaray, L.B. (2016). Riverscape genomics of a threatened fish across a hydroclimatically heterogeneous river basin. *Mol. Ecol.* 25, 5093-5113.

Colli, L., Joost, S., Negrini, R., Nicoloso, L., Crepaldi, P., Ajmone-Marsan P., and ECNOGENE Consortium. (2014). Assessing the spatial dependence of adaptive loci in 43 European and Western Asian goat breeds using AFLP markers. *PLoS One* 9, e86668.

De Kort, H., Vandepitte, K., Bruun, H.H., Closset-Kopp, D., Honnay, O., and Mergeay, J. (2014). Landscape genomics and a common garden trial reveal adaptive differentiation to temperature across Europe in the tree species *Alnus glutinosa*. *Mol. Ecol.* 23, 4709-4721.

Di Pierro, E.A., Mosca, E., Rocchini, D., Binelli, G., Neale, D.B., and La Porta, N. (2016). Climate-related adaptive genetic variation and population structure in natural stands of Norway spruce in the South-Eastern Alps. *Tree Genet*. *Genomes* 12, 16.

Eckert, A.J., Bower, A.D., Gonzalez-Martinez, S.C., Wegrzyn, J.L., Coop, G., and Neale, D.B. (2010). Back to nature: ecological genomics of loblolly pine (*Pinus taeda*, Pinaceae). *Mol. Ecol.* 19, 3789-3805.

Fitzpatrick, M.C., and Keller, S.R. (2015). Ecological genomics meets community-level modelling of biodiversity: mapping the genomic landscape of current and future environmental adaptation. *Ecol. Lett.* 18, 1-16.

Geraldes, A., Farzaneh, N., Grassa, C.J., McKown, A.D., Guy, R.D., Mansfield, S.D., Douglas, C.J., and Cronk, Q.C. (2014). Landscape genomics of *Populus trichocarpa*: the role of hybridization, limited gene flow, and natural selection in shaping patterns of population structure. *Evolution* 68, 3260-3280.

Hand, B.K., Muhlfeld, C.C., Wade, A.A., Kovach, R.P., Whited, D.C., Narum, S.R., Matala, A.P., Ackerman, M.W., Garner, B.A., Kimball, J.S., Stanford, J.A., and Luikart, G. (2016). Climate variables explain neutral and adaptive variation within salmonid metapopulations: the importance of replication in landscape genetics. *Mol. Ecol.* 25, 689-705.

Hecht, B.C., Matala, A.P., Hess, J.E., and Narum, S.R. (2015). Environmental adaptation in Chinook salmon (*Oncorhynchus tshawytscha*) throughout their North American range. *Mol. Ecol.* 24, 5573-5595.

Jones, M.R., Forester, B.R., Teufel, A.I., Adams, R.V., Anstett, D.N., Goodrich, B.A., Landguth, E.L., Joost, S., and Manel, S. (2013). Integrating landscape genomics and spatially explicit approaches to detect loci under selection in clinal populations. *Evolution* 67, 3455-3468.

Joost, S., Bonin, A., Bruford, M.W., Després, L., Conord, C., Erhardt, G., and Taberlet, P. (2007). A spatial analysis method (SAM) to detect candidate loci for selection: towards a landscape genomics approach to adaptation. *Mol. Ecol.* 16, 3955-3969.

Jordan, R., Dillon, S.K., Prober, S.M., and Hoffmann, A.A. (2016). Landscape genomics reveals altered genome wide diversity within revegetated stands of *Eucalyptus microcarpa* (Grey Box). *New Phytol.* 212, 992-1006.

Keller, S.R., Levsen, N., Olson, M.S., and Tiffin, P. (2012). Local adaptation in the flowering-time gene network of balsam poplar, *Populus balsamifera* L. *Mol. Biol. Evol.* 29, 3143-3152.

Leamy, L.J., Lee, C.R., Song, Q.J., Mujacic, I., Luo, Y., Chen, C.Y., Li, C.B., Kjemtrup, S., and Song, B.H. (2016). Environmental versus geographical effects on genomic variation in wild soybean (*Glycine soja*) across its native range in northeast Asia. *Ecol. Evol.* 6, 6332-6344.

Lee, C.R., and Mitchell-Olds, T. (2012). Environmental adaptation contributes to gene polymorphism across the *Arabidopsis thaliana* genome. *Mol. Biol. Evol.* 29, 3721-3728.

Lv, F.H., Agha, S., Kantanen, J., Colli, L., Stucki, S., Kijas, J.W., Joost, S., Li, M.H., and Marsan, P.A. (2014). Adaptations to climate-mediated selective pressures in sheep. *Mol. Biol. Evol.* 31, 3324-3343.

Manthey, J.D., and Moyle, R.G. (2015). Isolation by environment in White-breasted Nuthatches (S*itta carolinensis*) of the Madrean Archipelago sky islands: a landscape genomics approach. *Mol. Ecol.* 24, 3628-3638.

Mosca, E., Gugerli, F., Eckert, A.J., and Neale, D.B. (2016). Signatures of natural selection on *Pinus cembra*, and *P. mugo*, along elevational gradients in the Alps. *Tree Genet*. *Genomes* 12:9

Orsini, L., Mergeay, J., Vanoverbeke, J., and De Meester, L. (2013). The role of selection in driving landscape genomic structure of the waterflea *daphnia magna*. *Mol. Ecol.* 22, 583-601.

Pariset, L., Joost, S., Marsan, P.A., and Valentini, A. (2009). Landscape genomics and biased F_ST_ approaches reveal single nucleotide polymorphisms under selection in goat breeds of North-East Mediterranean. *BMC Genet.* 10, 7.

Parisod, C., and Christin, P.A. (2008). Genome-wide association to fine-scale ecological heterogeneity within a continuous population of *Biscutella laevigata* (Brassicaceae). *New Phytol.* 178, 436-447.

Pluess, A.R., Frank, A., Heiri, C., Lalagüe, H., Vendramin, G.G., and Oddoumuratorio, S. (2016). Genome-environment association study suggests local adaptation to climate at the regional scale in *Fagus sylvatica*. *New Phytol*. 210, 589-601.

Poncet, B.N., Herrmann, D., Gugerli, F., Taberlet, P., Holderegger, R., Gielly, L., Rloux, D., Thuiller, W., Aubert, S., and Manel, S. (2010). Tracking genes of ecological relevance using a genome scan in two independent regional population samples of *Arabis alpina*. *Mol. Ecol.* 19, 2896-2907.

Rellstab, C., Zoller, S., Walthert, L., Lesur, I., Pluess, A.E., Graf, R., Bodénès, C., Sperisen, C., Kremer, A.,and Gugerli, F. (2015). Signatures of local adaptation in candidate genes of oaks (*Quercus spp.*) with respect to present and future climatic conditions. *Mol. Ecol.* 25, 5907-5924.

Roffler, G.H., Amish, S.J., Smith, S., Cosart, T., Kardos, M., Schwartz, M.K., and Luikart, G. (2016). SNP discovery in candidate adaptive genes using exon capture in a free-ranging alpine ungulate. *Mol. Ecol. Resour.* 16, 1147-1164.

Rolland, J., Lavergne, S., and Manel, S. (2015). Combining niche modelling and landscape genetics to study local adaptation: A novel approach illustrated using alpine plants. *Perspect. Plant Ecol Evol. Syst.* 17, 491-499.

Roschanski, A.M., Csilléry, K., Liepelt, S., Oddou-Muratorio, S., Ziegenhagen, B., Huard, F., Ullrich, K.K., Postolache, D., Vendramin, G.G., and Fady, B. (2016). Evidence of divergent selection for drought and cold tolerance at landscape and local scales in *Abies alba* Mill. in the French Mediterranean Alps. *Mol. Ecol.* 25, 776-794.

Shryock, D.F., Havrilla, C.A., DeFalco, L., Esque, T.C., Custer, N., and Wood, T.E. (2015). Landscape genomics of *Sphaeralcea ambigua* in the Mojave Desert: a multivariate, spatially-explicit approach to guide ecological restoration. *Conserv. Genet.* 16, 1303-1317.

Stronen, A.V., Jędrzejewska, B., Pertoldi, C., Demontis, D., Randi, E., Niedziałkowska, M., Sidorovich, V.E., Kusak, J., Kojola, I., Karamanlidis, A.A., Ozolins, J., Dumenko, V., and Czarnomska, S.D. (2015). Genome-wide analyses suggest parallel selection for universal traits may eclipse local environmental selection in a highly mobile carnivore. *Ecol. Evol.* 5, 4410-4425.

Vangestel, C., Vázquez-Lobo, A., Martínez-García, P.J., Calic, I., Wegrzyn, J.L., and Neale, D.B. (2016). Patterns of neutral and adaptive genetic diversity across the natural range of sugar pine (*Pinus lambertiana, Dougl.*). *Tree Genet.Genomes* 12, 51.

Vincent, B., Dionne, M., Kent, M.P., Lien, S., and Bernatchez, L. (2013). Landscape genomics in Atlantic salmon (*Salmo salar*): searching for gene-environment interactions driving local adaptation. *Evolution* 67, 3469-3487.

Zhang, Y.H., Wang, I.J., Comes, H.P., Peng, H., and Qiu, Y.X. (2016). Contributions of historical and contemporary geographic and environmental factors to phylogeographic structure in a Tertiary relict species, *Emmenopterys henryi* (Rubiaceae). *Sci. Rep.* 6, 24041.
